# The association between being breastfed in infancy and risks of cancer in adulthood—a UK Biobank study

**DOI:** 10.1038/s44276-024-00061-x

**Published:** 2024-05-14

**Authors:** Dan Hameiri-Bowen, Dorthe C. Pedersen, Britt W. Jensen, Julie Aarestrup, Kathleen M. Rasmussen, Jennifer L. Baker, Lise G. Bjerregaard

**Affiliations:** 1 Center for Clinical Research and Prevention, Copenhagen University Hospital—Bispebjerg and Frederiksberg, Copenhagen, Denmark; 2 Division of Nutritional Sciences, Cornell University, Ithaca, NY USA

## Abstract

**Background:**

Being breastfed has established benefits for infant health, but its long-term effects on adult diseases, including cancer, remain underexplored. We examined associations between being breastfed in infancy and the risks of common cancers.

**Methods:**

Data from 339,115 participants (191,117 women) enrolled in the UK Biobank with self-reported breastfeeding data were linked to national cancer registries. Cox models estimated sex-specific hazard ratios (HR) and 95% confidence intervals (CI) for the associations between being breastfed (ever/never) and risks of overall cancer as well as common cancer sites.

**Results:**

In total, 34,392 incident cancers (17,895 in women) were registered. The HR of overall cancer associated with being breastfed was 1.05 (95% CI 1.01–1.09) in women and 1.00 (95% CI 0.96–1-04) in men. In site-specific analysis being breastfed was associated with an increased risk of breast and ovarian cancer in women and a reduced risk of oesophageal cancer in men.

**Discussion:**

We found that having been breastfed was associated with a marginally increased risk of adult cancer in women, but we found no evidence of an association in men. These findings should be viewed within the study limitations, and do not outweigh the many benefits that breastfeeding provides.

## Introduction

Cancer is a leading cause of morbidity and premature mortality worldwide [1]. Despite regional heterogeneity, global cancer incidence is rising [1, 2]. These increases likely reflect demographic shifts, and improvements in cancer screening and detection, alongside changes in the distribution of modifiable risk factors and, potentially, changes in breastfeeding [3]. Breastfeeding provides substantial short and long-term health benefits for both the mother and child [4]. Compared to infants who are not breastfed, breastfed infants have lower morbidity from infectious disease, score higher in intelligence tests, and may be protected against overweight and diabetes in later life [5]. Despite these benefits, breastfeeding rates in both the United Kingdom (UK) and Europe remain substantially below the World Health Organization’s (WHO) recommendations to initiate breastfeeding within the first hour of birth and maintain exclusive breastfeeding for the first six months of life [4, 6, 7]. In the UK, the proportion of infants who were ever breastfed dropped from 80% to 60% between 1934 and 1970 [8, 9]. Estimates suggest that in the early 1990s, fewer than 1% of UK infants received 6 months of exclusive breastfeeding and that despite improvements in breastfeeding initiation, this was still the case in 2010 [7, 10].

Despite the well-described benefits of having been breastfed, associations with cancer risks are less well-described. Having been breastfed has been associated with a reduced risk of childhood cancer, including leukaemia and neuroblastomas [11]. Although adult cancers often differ from childhood cancers in underlying pathology and behaviour, the fundamental processes of malignant change likely share common features [12]. Consequently, there is reason to examine whether having been breastfed also protects against adult cancers. Current evidence of associations between having been breastfed and adult cancer risk is scarce and inconsistent. In the largest study to date, the UK-based Million Women Study, an increased risk of colorectal cancer among women who had been breastfed was described [13]. Other studies have observed an inverse association between having been breastfed and pre-menopausal breast cancer, but not other cancer sites [14]. Notably, a substantial knowledge gap remains for evidence of associations of having been breastfed with cancer in men, and for studies powered to detect associations at separate cancer sites.

Having been breastfed as an infant may be linked to adult cancer risks through its associations with obesity and inflammation, which are established risk factors for many cancer forms [15–17]. For example, having been breastfed is associated with appetite regulation, energy balance and metabolism throughout life [17, 18]. These associations may affect the development of risk factors for cancer such as childhood growth patterns, child and adult obesity and in turn obesity-related cancers [19, 20]. Breastfeeding may also be linked to adult cancer risks through effects on the early-life immune system [21]. Breastmilk is rich in bioactive components essential for both protection from environmental pathogens and the development of the initially insufficient, immature and ineffective infant immune system [22]. Connected to its immunological properties, being breastfed is associated with the pattern of microbial colonisation in the child, which persists into later life, and could affect cancer risk [23–25].

Consequently, we hypothesised that having been breastfed may be associated with reduced cancer risk in later life. To assess this, we utilised data from the UK Biobank to examine sex-specific associations between self-reported breastfeeding status and the risk of adult cancer overall. Following this, we examined the associations with the 10 most common cancer forms in each sex to further describe potential site-specific effects.

## Methods

### Data resource and study population

The UK Biobank is a prospective cohort study that enrolled participants at ages 40–49 years. A full study protocol is described online [26]. In brief, between 2006–2010, participants were invited to an assessment centre at one of 21 sites across the UK [27]. At the assessment centres, participants completed a touchscreen questionnaire providing information on early life, socio-demographic, lifestyle, environmental, and reproductive factors. Anthropometric measurements were taken by technicians following standardised protocols [26]. Breastfeeding data were collected with the question “Were you breastfed when you were a baby” (yes/no/do not know/prefer not to answer).

All information was collected at the recruitment centre. Variables requested from the data resource included if the participants’ mother smoked (yes/no), participant year of birth, ethnicity (re-categorised into Black, Asian, White, mixed, other), birthweight (kg), relative body size at age 10 (smaller, about average, larger), the participants’ educational level achieved (recategorized into low, intermediate, high), location of the assessment centre, participant age at enrolment (years), body mass index (BMI; kg/m^2^) at enrolment, smoking status (never-smoked, ex-smoker, current smoker), hormone replacement therapy use (ever vs. never used) and Townsend Deprivation score (numerical measure of relative material deprivation at study entry assigned based on national census output areas).

Biobank data were electronically linked to the UK’s national cancer and death registries. These registers contain data starting from the early 1970s [28]. Cancer forms were defined by International Classification of Diseases 10th edition (ICD-10) codes and the 10 most common incident cancer forms in each sex were retained for exploratory site-specific analysis (Supplementary Table 1). Where non-sex-specific cancers appeared in the top 10 of only one sex, they were assessed in both sexes to facilitate comparisons. Following this criteria, 13 cancers were included (cancer of the bladder, breast, colon, endometrium, kidney, lung, malignant melanomas of the skin, non-Hodgkin’s lymphoma, oesophagus, ovaries, pancreas, prostate, and rectum). Models for overall cancer included all malignant neoplasms (ICD-10 codes: C00–C97). Non-melanoma skin cancer was not included in any analysis. Participants with non-melanoma skin cancer were either included in the non-cancer group or, if diagnosed with a second cancer form, they contributed to the analyses of this cancer form. Only incident cancer cases (from the date of enrolment into UK Biobank) were included.

### Study population

**Fig. 1:**
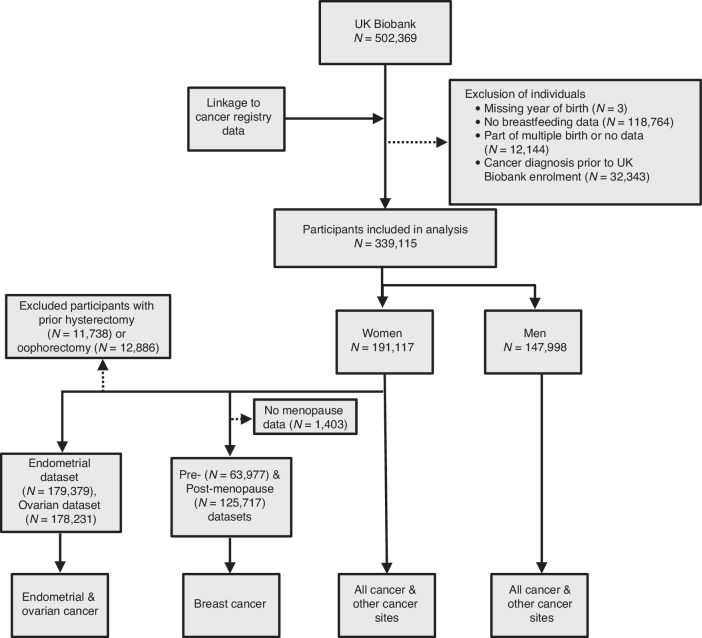
Flow chart of eligible individuals and those included in the study at each stage and within each separate analysis.

Participants were excluded from all analyses if they answered, “do not know”, “prefer not to answer” or had missing data for if they were breastfed as an infant. Participants who had missing information for or who reported being part of a multiple births or adopted were also excluded (Fig. 1). For the analyses of endometrial and ovarian cancer, women with self-reported hysterectomy or oophorectomy were excluded from the analysis, respectively. Participants with their first cancer diagnosis before enrolment into the UK Biobank, other than non-melanoma skin cancer (C44), hydatidiform moles (O01), benign tumours (D10-D36), or in situ neoplasms (D00-D09), were excluded.

### Statistical analyses

Participant characteristics were described as percentages within a given category or median and interquartile range (IQR) by breastfeeding status. Where present, missing data for each variable were categorised as a new “missing” category. The characteristics of those with and without breastfeeding data were also compared. The occurrence of each cancer form was counted by breastfeeding status and sex. To examine if having been breastfed as an infant was associated with cancer risk in later life, associations between having been breastfed and incident cancer overall, alongside site-specific cancers were examined using sex-specific Cox proportional hazard models with age as the underlying time scale. Sex-specific models were constructed owing to the considerable sex differences in cancer susceptibility between sexes [29]. In the constructed models, follow-up began at the date of enrolment into the UK Biobank and ended at the date of first cancer, death or at the end of follow-up in the linked cancer registry (25th June 2021), whichever came first.

Potential confounders of the association between having been breastfed and cancer risks were identified based on availability in the UK Biobank and *a priori*, literature-driven hypotheses. Maternal smoking and participant ethnicity were considered confounders and were included as covariates in adjusted models. For the analyses of breast and endometrial cancers, we included self-reported use of hormone replacement therapy as an additional covariate. Cox models were stratified by age category at enrolment (38–44, 45–49, 50–54, 55–59, 60–64, 65–73 years), year of birth, and location of the assessment centre. Both crude and adjusted hazard ratios (HR) and their respective 95% confidence intervals (CI) are reported. For the reader’s interest, *p* values for each model are presented in the supplementary materials. Although identified as a potential confounder, birthweight had a high level of missingness (> 30% compared with < 5% for other included variables) and was therefore not included as a covariate.

Models for breast cancer were divided into pre-menopausal and post-menopausal periods. In the pre-menopause model, women who did not report having experienced menopause contributed person years of follow-up from age at assessment to 55 years of age. In the post-menopausal analysis, women contributed person years of follow-up from age 55 years or age at enrolment if they self-reported having experienced menopause at the assessment. Fifty-five years of age was selected as the cut-off for menopause as in a wide range of international studies, almost all women had reached menopause by this age [30]. Women who self-reported having not had menopause but were over the age of 55 were excluded from the post-menopausal analysis (*n* = 1317).

To account for the number of outcomes analysed in the exploratory site-specific analysis, we presented the Bonferroni corrected site-specific family error rate (α). The adjustment factor for this correction was determined by the number of sites analysed for each sex.

To assess the proportionality of hazards over time, potential differences in associations between having been breastfed and cancer risks by categories of age at risk (38–58, 59–62, 63–66, 67–73, years) were tested in nested models with and without cross-product terms. Age categories were selected to approximate an equal number of participants in each category. Similarly, potential interactions with birth cohorts (1934–1944, 1945–1949, 1950–1954, 1955–1959, 1960–1971) were tested in nested models with and without cross-product terms.

All analyses were performed in R (Version 4.1.0). Code will be made available on request to the corresponding author. This project was registered with the UK Biobank on 19-10-2022 (approved research ID: 95095) and approved by the Danish Data Protection Agency (P-2022-445).

## Results

From a total of 502,369 UK Biobank participants, 339,115 matched the inclusion criteria and were included in this study (Fig. 1). Characteristics of participants with and without breastfeeding data are presented in Supplementary Table 2. Participants without breastfeeding data were older, had more missing data and had lower birthweight than those with breastfeeding data. Of the participants with breastfeeding data, 191,117 (56.4%) were women. Most participants (247,404 [73%]) responded “yes” to having been breastfed as a baby. Participants who reported having been breastfed were born earlier, were older, less socioeconomically deprived at enrolment, and were more likely to be male when compared to those who reported having not been breastfed (Table 1).

**Table 1:** Characteristics of the study population by breastfeeding status

Characteristic	Breastfeeding data available (*n* = 339,115)	
	Breastfed (*n* = 247,397)	Not breastfed (*n* = 91,718)
Female sex, *n* (%)	135,060 (54.6%)	56,057 (61.1%)
Ethnicity, *n* (%)		
White	227,406 (92.0%)	89,389 (97%)
Asian	7500 (3.0%)	578 (0.6%)
Black	5960 (2.4%)	297 (0.3%)
Mixed	1453 (0.6%)	578 (0.6%)
Other	4130 (1.7%)	655 (0.7%)
Missing	948 (0.4%)	221 (0.2%)
**Early life factors**		
Year of birth category		
<1945	63,742 (25.8%)	14,538 (15.9%)
1945–1949	58,972 (23.8%)	16,607 (18.1%)
1950–1954	44,830 (18.1%)	14,977 (16.3%)
1954–1959	37,567 (15.2%)	16,590 (18.1%)
≥1960	42,286 (17.1%)	29,006 (31.6%)
Birthweight in grams, *n* (%)		
≤2750	19,897 (8.0%)	12,181 (13.3%)
>2750–3250	44,768 (18.1%)	19,274 (21.0%)
>3250–3750	55,118 (22.3%)	21,300 (23.2%)
>3750–4250	22,352 (9.0%)	8254 (9.0%)
>4250–4750	8938 (3.6%)	3218 (3.5%)
>4750	2709 (1.1%)	873 (1.0%)
Missing	93,615 (37.8%)	26,618 (29.0%)
Mother smoked (%)		
Yes	56,249 (22.7%)	29,510 (32.2%)
No	167,017 (67.5%)	53,198 (58%)
Missing	24,131 (9.8%)	9010 (9.8%)
Relative size at age 10, *n* (%)		
Thinner	79,644 (32.2%)	30,406 (33.2%)
About Average	125,900 (50.9%)	43,826 (47.8%)
Larger	37,956 (15.3%)	16,258 (17.7%)
Missing	3897 (1.6%)	1228 (1.3%)
**Characteristics at recruitment**		
Age at recruitment (median (IQR)	58 (50, 63)	53 (46, 60)
Townsend deprivation score, median (IQR)*^a^	−2.17 (−3.67 –0.46)	−2.08 (−3.61 −0.63)
Education, *n* (%)		
High	116,969 (47.3%)	38,630 (42.2%)
Intermediate	91,047 (36.8%)	38,036 (41.5%)
Low	35,177 (14.2%)	13,652 (14.9%)
Missing	4204 (1.7%)	1400 (1.5%)
Smoking status, *n* (%)		
Never-smoked	137,018 (55.4%)	52,886 (57.7%)
Ex-smoker	85,071 (34.4%)	28,312 (30.9%)
Current smoker	24,478 (9.9%)	10,269 (11.2%)
Missing	830 (0.3%)	251 (0.3%)
Body mass index, kg/m^2^ (median, (IQR))^*b^	26.7 (24.1–29.8)	26.6 (24.0–29.9)
Hormone replacement therapy*^c^		
Yes	50,676 (37.5%	17,406 (31.1%)
No	83,834 (62.1%)	38,487 (68.7%)
Missing	550 (0.4%)	164 (0.2%)

*IQR* interquartile range. Where participant data were entirely missing or the participant answered, “Do not know” or “Prefer not to answer”, data were recoded as “Missing”.

^*a^A total of 434 individuals had missing Townsend deprivation score data. The more negative the deprivation score the less socioeconomically deprived the participant is.

^*b^1771 participants had missing body mass index data.

^*c^Hormone replacement therapy was only assessed in female participants.

During a median follow-up of 12.2 years (IQR = 11.4–13.0), 17,865 and 16,497 first-incident cancers were recorded in women and men, respectively. The number of cases at different cancer sites by breastfeeding status and sex are provided in Table 2.

**Table 2:** Cancer cases by breastfeeding status and sex.

Cancer Site	Women (*n* = 191,117)			Men (*n* = 147,998)			
	Breastfed (*n* = 135,060 participants)	Not breastfed (*n* = 56,057 participants)	Total cases	Breastfed *n* = 112,337 participants	Not Breastfed *n* = 35,661 participants	Total cases	
Overall cancer^*a^	13,124	4741	17,865	13,230	3267		16,497
Pre-menopausal breast^*b^	942	595	1537	—	—		—
Post-Menopausal Breast^*c^	4055	1179	5234	—	—		—
Endometrium^*d^	795	266	1061	—	—		—
Ovary^*d^	521	164	685	—	—		—
Prostate	—	—	—	5548	1,252		6800
Colon	933	297	1230	956	249		1205
Lung	767	276	1043	895	212		1107
Malignant Melanoma of the Skin	704	273	977	690	191		881
Rectum	362	128	490	598	140		738
Non-Hodgkin’s Lymphoma	507	161	668	541	139		680
Kidney	238	83	321	411	117		528
Pancreas	279	98	377	321	67		388
Bladder	121	46	167	391	91		482
Oesophagus	132	55	187	317	107		424

^*a^The sum of listed cancers will not sum to overall cancer as cancers outside the top 10 contribute to this number.

*^b^For pre-menopausal breast cancer the population size was 31,160 breastfed and 24,817 not breastfed.

*^c^For post-menopausal breast cancer the population size was 30,855 breastfed and 98,862 not breastfed.

*^d^Owing to exclusions the population size was 136,326 breastfed and 53,053 not breastfed for endometrial cancer. The population size for ovarian cancer was 125,848 breastfed and 52,383 not breastfed.

**Fig. 2:**
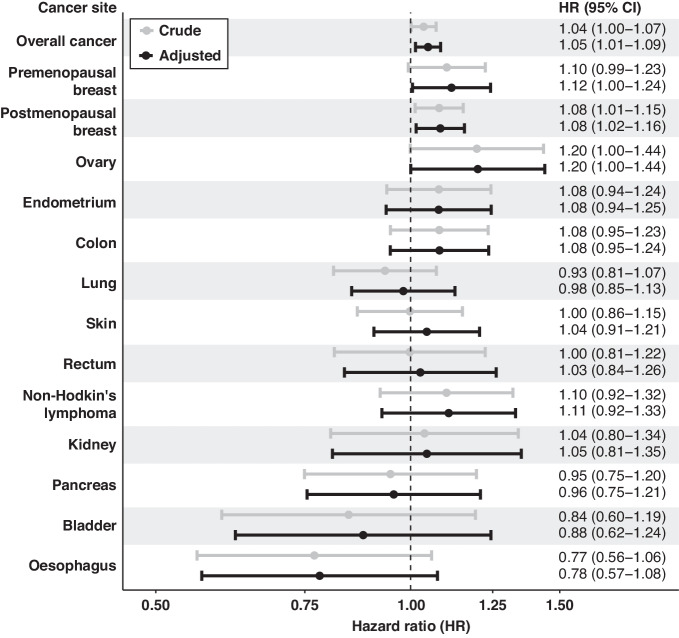
Forest plot of associations between being breastfed as an infant and cancer risk in women. Crude models are in grey and adjusted models (adjusted for ethnicity and maternal smoking status) are in black. All models are stratified by year of birth, centre location, and age group at enrolment.

**Fig. 3:**
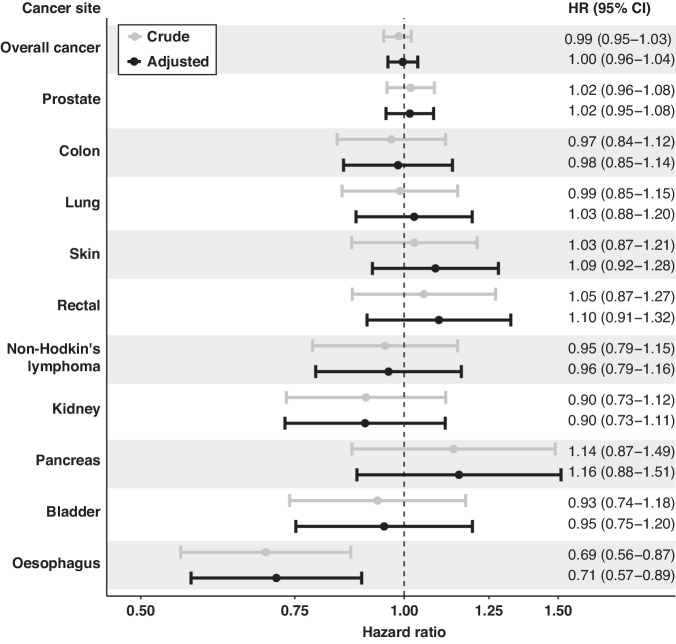
Forest plot of associations between being breastfed as an infant and cancer risk in men. Crude models are in grey and adjusted models (adjusted for ethnicity and maternal smoking status) are in black. All models are stratified by year of birth, centre location, and age group at enrolment.

Having been breastfed as an infant was associated with a slightly increased risk of overall cancer in women (HR = 1.05 [95% CI, 1.01–1.09]) (Fig. 2). Among men, there was no evidence that having been breastfed was associated with overall cancer (HR = 1.00 [95% CI, 0.96–1.04]) (Fig. 3). In exploratory site-specific analyses, no associations were observed for 10 of the 13 sites assessed in women. Having been breastfed was associated with an increased risk of pre-menopausal breast cancer (HR = 1.12 [95% CI, 1.00–1.24]), post-menopausal breast cancer (HR = 1.08 [95% CI, 1.02–1.16]) and ovarian cancer (HR = 1.20 [95% CI, 1.00–1.44]) compared to those who were not breastfed (Fig. 2, Supplementary Table 3). The inclusion of the use of hormone replacement therapy in the models for breast and endometrial cancer minimally affected the associations described (Supplementary Table 3). For site-specific analyses in women, after correction for multiple testing, no *p*-value was below the Bonferroni-adjusted family error rate α (0.0042 [0.05/12]) (Supplementary Table 3). In men, no site-specific associations were observed for 9 of the 10 cancer sites assessed. Men who were breastfed as infants had a reduced risk of oesophageal cancer (HR = 0.71 [95% CI, 0.57–0.89]) (Fig. 3). The *p*-value for oesophageal cancer in men was below the Bonferroni-adjusted family error rate *α* of 0.005 [0.05/10]) (Supplementary Table 3).

No clear increasing or decreasing birth-cohort effects were observed for the majority of cancers. Birth-cohort effects were observed for kidney cancer in women and lung and skin cancer in men (Supplementary Table 4). Violations of the proportional hazards assumptions were observed for kidney cancer and non-Hodgkin’s lymphoma in women. (Supplementary Fig. 1). There was no evidence of proportional hazard assumption violations in men.

## Discussion

In this study, based on a large UK population, having been breastfed was associated with a slightly increased risk of overall cancer in women, but no evidence of an association was observed in men. An exploratory analysis across common cancer sites provided weak evidence that these findings may be driven by an increased risk of breast and ovarian cancer in women who were breastfed. In men, site-specific models identified a reduced risk of oesophageal cancer in men who were breastfed. Notably, the overall association in women is driven by sex-specific hormonal cancer forms, potentially explaining the sex differences observed in the overall cancer estimates. These findings should be interpreted cautiously owing to the limitations of the study.

We initially hypothesised that the association of breastfeeding with healthy growth, development and reduced risk of childhood obesity would protect breastfed infants from cancer in later life. The increased risk of overall cancer in women who were breastfed is the opposite of the protective effect we expected. One possible explanatory hypothesis is that the increased risk of cancer could have been related to the timing of weight gain and how this is associated with breastfeeding [20]. Early-life BMI has been inversely associated with the risk of breast cancer, independently of adult BMI [31, 32], and breastfed children have a lower BMI compared to those who are not breastfed. Consequently, breastfed women may have an increased risk of breast cancer in adulthood, compared to those who are not breastfed, owing to their lower early-life BMI. Despite this, we would expect adult BMI to be more important for adult cancer risk as a less time-distanced exposure. Furthermore, the inverse association of breastfeeding with BMI may not track fully into adulthood, but more evidence is required to understand the relationship between breastfeeding and adult BMI [33]. Moreover, the association between breastfeeding and early-life BMI relies on individuals being breastfed long and intensely enough to affect growth outcomes. The exposure variable (ever vs. never breastfed) available in this data cannot capture these important aspects of breastfeeding. Consequently, from the data available, we cannot conclude that the increased risk of cancer is driven by reduced childhood BMI in breastfed infants. Furthermore, and contrary to the hypothesis that the increased risk of cancer in women having been breastfed is explained through inverse associations with body size, childhood overweight and higher BMI have been shown to be positively associated with the risk of ovarian cancer [34]. The differences in the direction of the relationship between childhood BMI and these two cancer sites serve to highlight the difficulty of linking the effect of breastfeeding on early-life growth to subsequent cancer risk and reflect the complex web of early-life exposures that may influence later cancer risk [35].

The increased risk we observed between having been breastfed and both breast and ovarian cancer is not consistent with the previous literature. Both the Million Women study (*n* = 395,363) and an analysis of 89,385 pre-menopausal and 50,586 post-menopausal American women born between 1921 and 1965 and recruited into one of the two Nurses Health Studies described no evidence of an association between having been breastfed (ever vs. never breastfed) and either breast or ovarian cancer [13, 36]. In the Million Women Study, findings across other cancer sites (endometrial, lung, non-Hodgkin’s lymphoma, oesophageal, pancreatic, skin, and stomach cancer) were consistent with our findings [13]. The authors did report an increased risk of colon and rectal cancer (relative risk (RR) = 1.19 [95% CI, 1.12–1.25], RR = 1.14 [95% CI, 1.02–1.26], respectively), which was not replicated in our study. It is plausible that differences in the study populations may explain these differences. For example, women recruited into the Million Women Study were born earlier and were older on average than the UK Biobank participants, but the exact reasons for intra-study variation across cancer sites are challenging to explain.

In our study, there was no evidence of an association between having been breastfed and overall cancer in men as well as for 9 out of 10 cancer sites examined. We did observe a relatively strong inverse association between being breastfed and the risk of oesophageal cancer. Interestingly, although sex differences in oesophageal cancer are widely reported and thought to be driven by hormonal and genomic factors, the measure of association for women in this study (HR = 0.78 [95% CI, 0.57–1.08]) and the Million Women Study (crude RR = 0.87 [95% CI, 0.76–0.99]) suggest that breastfed women might also have been protected from oesophageal cancer [13]. In our study, the wider confidence intervals for the association with oesophageal cancer reflected a lower oesophageal cancer incidence in women, and although the association reported in the Million Women Study was attenuated when covariates were included (many of which were likely to mediate the association), there appears to be evidence of a signal [13]. Although it is hard to hypothesise why breastfeeding would have a protective association with only oesophageal cancer, which shares common risk factors with many other cancer sites, it would be interesting to see if this association is replicated in future studies.

The strengths of this study include the large sample size and linkage of UK Biobank participants to the high-quality and high-coverage National Health Service cancer registries. Although UK Biobank participants are healthier than the general population, the size of the resource and the number of cancer cases likely provide a good assessment of aetiological associations between having been breastfed and cancer risk, which can be generalised [37]. The size of the cohort and long-term follow-up also facilitated assessment across many cancer sites in women and men separately, which few resources are capable of facilitating.

This study has important limitations. The precision in the measure of the exposure is limited owing to the categorising of breastfeeding into “ever” or “never” groups. This fails to account for individual heterogeneity in the duration, intensity, and exclusivity of breastfeeding. Detailed information on breastfeeding would allow a better assessment of how this may be associated with cancer, including investigation of dose-dependent associations and insight into the optimal duration of breastfeeding. The dichotomous nature of the exposure variable limits our interpretation as we are unable to examine a quantitative exposure to breastmilk. As we hypothesise that breastfeeding influences cancer risk through established cancer risk factors, the amount and length of exposure to breastfeeding are essential to describe. The measurement of the exposure variable is also a limitation of this study because the collection of breastfeeding data by participant recall potentially introduces non-differential misclassification bias, thus, we cannot preclude a potential underestimation of the results. Nevertheless, previous studies have described high levels of concordance between prospectively collected and recalled data on ever breastfeeding (yes/no), although agreement is higher if individuals are breastfed for at least 1 month [38].

The relatively large number of excluded individuals with missing breastfeeding data is a further limitation. We suggest that differences between those with and without breastfeeding data are driven by the missing data population being born earlier. As such, we cannot preclude some selection bias in the study. Despite this, those with breastfeeding data still represented a broad range of birth years. Among these, we found few interactions between breastfeeding and the birth-cohort in the associations with cancer, and thus, we find it unlikely that our analysis underestimates the effect of breastfeeding. For individual cancer sites with birth-cohort effect, they were limited and showed no consistent pattern or replication across cancer sites. Consequently, we conclude that the described birth-cohort effects were spurious and resulted from low numbers of cancers, which was reflected in the wide confidence intervals.

The associations between breastfeeding and many factors across the life course present methodological challenges, such as the identification of potential confounders. A critical data gap in the UK Biobank is the lack of information on maternal factors, such as maternal socioeconomic position, age, and obesity, which could potentially confound the observed associations, alongside influencing the duration and intensity of breastfeeding [36]. Models constructed also did not include birthweight as a potential confounder, as it was poorly recorded in the available data. Consequently, there is a risk of bias due to not controlling for birthweight. Moreover, the models did not include later life determinants of cancer, such as the family history of cancer, adult body size and body composition, socioeconomic factors, diet, or physical activity since these factors are mediators rather than confounders, and it was not the aim of this study to assess mediation through these factors. Other potentially important data such as information on complementary feeding or infant diet were not collected and are likely to have changed over the four decades over which the participants were born. This absence of this data restricts the interpretation of the results and relevance to contemporary populations where the availability of breastfeeding alternatives, as well as societal pressure and norms, will markedly differ.

There are also limitations to our interpretation of the findings resulting from the design of this study. As a result of the age profile of participants in the UK Biobank, we are unable to extend our findings to lifetime cancer risk, especially for cancers with earlier ages of onset. Examination of these associations in a younger population would be an important addition to future studies. The UK Biobank is susceptible to survival bias as participants had to survive into middle age to be included. If having been breastfed is associated with cancer or survival before enrolment, which we expect, these effects could lead to under- or over-estimation of the effect sizes. We also investigated associations with many cancer forms, increasing the family-wise error rate. The cancer site-specific associations have been interpreted conservatively as these could be spurious findings that require further validation.

In conclusion, we found limited evidence of an association between having been breastfed and adult cancer risk in men and women. Having been breastfed was associated with a marginally increased risk of adult cancer in women, but no evidence of an association was reported in men. Site-specific analyses indicated that women who were breastfed had an increased risk of both breast and ovarian cancer, although these associations were not maintained after adjustment for multiple tests. Men who were breastfed as infants had a reduced risk of oesophageal cancer. For the interpretation of these results, we stress that the deleterious effects reported here are far outweighed by the immeasurable benefits of being breastfed for both mother and child. As the UK and European countries struggle to achieve the WHO recommendations on breastfeeding, these findings should not undermine efforts to create the conditions for initiation of and sustained breastfeeding.

## Supplementary information

Supplementary Tables Figures

## Data Availability

All data is publicly available from the UK Biobank. All code used for the completion of this manuscript will be made available as highlighted in the text.
