# Supplementary material for: The association between being breastfed in infancy and risks of cancer in adulthood—a UK Biobank study
Source: BJC Rep. 2024 May 14;2:40. doi: 10.1038/s44276-024-00061-x (PMC11523963; doi:10.1038/s44276-024-00061-x)
Supplement: Supplementary file 1 — Supplementary Tables Figures [file 44276_2024_61_MOESM1_ESM.docx]

**Supplementary Table 1:** Cancer definitions by cancer site

| **Cancer Site** | **ICD-10 Codes included** |
| --- | --- |
| Overall Cancer | C00-C97, D05  C44 (non-melanoma skin cancer) excluded |
| Oesophagus | C15-C15.9 Malignant neoplasm of the oesophagus |
| Colon | C18 – C18.9 Malignant neoplasm of the colon |
| Rectal | C19 – C19.9 Malignant neoplasms of the rectosigmoid junction  C20 – C20.9 Malignant neoplasm of the rectum |
| Pancreas | C25 – C25.9 Malignant neoplasms of the pancreas |
| Lung | C34 – C34.9 Malignant neoplasm of the bronchus and lung  C45 – C45.9 Mesothelioma |
| Skin | C43-C43.9 Malignant melanoma of skin |
| Breast | C50 – C50.9 Malignant neoplasm of the breast  D05 – D05.9 Carcinoma in situ of the breast |
| Endometrium | C54 Malignant neoplasms of the corpus uteri  C55 Malignant neoplasms of the uterus (Part unspecified) |
| Ovary | C56 Malignant neoplasm of the ovary |
| Prostate | C61 Malignant neoplasm of the prostate |
| Kidney | C64 Malignant neoplasm of the kidney, except renal pelvis |
| Bladder | C67-C69.9 Malignant neoplasm of the bladder |
| Diffuse non-Hodgkin’s lymphoma | C82-C82.9 Follicular (nodular) non-Hodgkin’s lymphoma  C83-C83.9 Diffuse non-Hodgkin’s lymphoma  C84-C84.9 Peripheral and cutaneous T-cell lymphomas  C85-C85.9 Other and unspecified types of non-Hodgkin’s lymphoma  C96-C96.9 Other and unspecified malignant neoplasms of lymphoid, haematopoietic, and related tissue |

**Supplementary Table 2:** Characteristics of individuals with breastfeeding and without breastfeeding data

| **Characteristic** | **UK Biobank Population (n = 502,369^a^)** | |
| --- | --- | --- |
|  | **Breastfeeding Data Absent (n = 118,764)** | **Breastfeeding Data Present (n = 383,602^b^)** |
| **Female Sex, n (%)** | 52,652 (44%) | 220,648 (58%) |
| **Ethnicity, n (%)** | | |
| White | 113,094 (95%) | 359,478 (94%) |
| Asian | 1,317 (1.1%) | 8,519 (2.2%) |
| Black | 1,101 (0.9%) | 6,834 (1.8%) |
| Mixed | 669 (0.6%) | 2,284 (1.3%) |
| Other | 955 (0.8%) | 5,173 (1.3%) |
| Missing | 1,628 (1.4%) | 1314 (0.3%) |
| **Early Life Factors** |  |  |
| Year of birth category |  |  |
| <1945 | 44,823 (38%) | 93,098 (24%) |
| 1945-1949 | 30,732 (26%) | 86,479 (23%) |
| 1950-1954 | 17,134 (14%) | 66,931 (17%) |
| 1954-1959 | 12,615 (11%) | 59,666 (16%) |
| ≥1960 | 13,460 (11%) | 77,428(20%) |
| Birthweight in grams, n (%) |  |  |
| ≤2750 | 6,703 (5.6%) | 44,033 (10%) |
| >2750-3250 | 7,839 (6.6%) | 71,937 (19%) |
| >3250-3750 | 8,198 (6.9%) | 84,422 (22%) |
| >3750–4250 | 3,735 (3.1%) | 33,699 (8.8%) |
| >4250–4750 | 2,156 (1.8%) | 13,414 (1.0%) |
| >4750 | 810 (0.7%) | 3,961 (1.0%) |
| Missing | 89,323 (75%) | 136,136 (35%) |
| Mother smoked (%) |  |  |
| Yes | 30,191 (25%) | 96,388 (25%) |
| No | 59,822 (50%) | 246,349 (64%) |
| Missing | 28,751 (24%) | 40,865 (11%) |
| Relative size at age 10, n (%) |  |  |
| Thinner | 38,563 (32%) | 125,046 (33%) |
| About Average | 57,629 (49%) | 191,931 (50%) |
| Larger | 17,317 (15%) | 60,832 (16%) |
| Missing | 5,255 (4.4%) | 5,793 (1.5%) |
| **Characteristics at Recruitment** | | |
| Age at recruitment (Median (IQR) | 61 (54, 65) | 57 (49, 62) |
| Townsend Deprivation Score, Median (IQR) | -2.08 (-3.6, 0.70) | -2.15 (-3.66, 0.51) |
| Missing | 147 (0.1%) | 476 (0.1%) |
| Education, n (%) | | |
| High | 45,006 (38%) | 174,618 (46%) |
| Intermediate | 41,597 (35%) | 145,759 (38%) |
| Low | 28,397 (24%) | 56,860 (15%) |
| Missing | 3,764 (3.2%) | 6,365 (1.7%) |
| Smoking Status, n (%) | | |
| Never smoked | 60,278 (51%) | 213,171 (56%) |
| Ex-smoker | 43,323 (36%) | 129,685 (34%) |
| Current Smoker | 13,460 (11%) | 39,501 (10%) |
| Missing | 1703 (1.4%) | 1,245 (0.3%) |
| Body Mass Index, kg/m^2^ (Median, (IQR)) | 27.0 (24.5, 30.1) | 26.6 (24.0, 29.8) |
| Missing | 1,116 (0.9%) | 1,988 (0.5%) |
| Hormone Replacement Therapy^c^ | | |
| Yes | 23,798 (45%) | 80,104 (36%) |
| No | 28,084 (53%) | 139,740 (63%) |
| Missing | 770 (1.5%) | 804 (0.4%) |

IQR = Inter-quartile range. Where participant data were entirely missing or the participant answered, “Do not know” or “Prefer not to answer”, data were recoded as “Missing. *^a^ The number analysed was 502,366 as three individuals did not have year of birth data and were dropped from this table *^b^ The number with breastfeeding information is greater than the study table as further exclusion criteria were applied to the population with breastfeeding data. *^c^Hormone replacement therapy was only assessed in women.

**Supplementary Table 3**: Associations between being breastfed as an infant and hazard ratios for cancer sites unadjusted and adjusted for ethnicity and maternal smoking. The models are stratified by age group at enrolment, location, and year of birth.

|  | **Women** | | **Men** | |
| --- | --- | --- | --- | --- |
| **Cancer Site** | **Crude HR (95% CI)** | **Adjusted HR (95% CI)** | **Crude HR (95% CI)** | **Adjusted HR (95% CI)** |
| **Overall Cancer** | 1.04 (1.00-1.07), *p* = 0.04 | 1.05 (1.01-1.09), *p* = 0.01 | 0.99 (0.95-1.03), *p* = 0.47 | 1.00 (0.96-1.04), *p* = 0.86 |
| **Pre-Menopausal Breast***^a^ | 1.10 (0.99-1.23), *p* = 0.07 | 1.12 (1.00-1.24), *p* = 0.04 | - | - |
| **Post-Menopausal Breast** *^a^ | 1.08 (1.01-1.15), *p* = 0.02 | 1.08 (1.02-1.16), *p* = 0.02^1^ | - | - |
| **Prostate** |  |  | 1.02 (0.96-1.08), *p* = 0.59 | 1.02 (0.95-1.08), *p* = 0.64 |
| **Ovary** | 1.20 (1.00-1.44), *p* = 0.05 | 1.20 (1.00-1.44), *p* = 0.05 | - | - |
| **Endometrium***^a^ | 1.08 (0.94-1.24), *p* = 0.29 | 1.08 (0.94-1.25), *p* = 0.29^1^ | - | - |
| **Colon** | 1.08 (0.95-1.23), *p* = 0.25 | 1.08 (0.95-1.24), *p* = 0.25 | 0.97 (0.84-1.12), *p* = 0.64 | 0.98 (0.85-1.14), *p* = 0.83 |
| **Lung** | 0.93 (0.81-1.07), *p* = 0.33 | 0.98 (0.85-1.13), *p* = 0.78 | 0.99 (0.85-1.15), *p* = 0.89 | 1.03 (0.88-1.20), *p* = 0.74 |
| **Malignant Melanomas of the Skin** | 1.00 (0.86-1.15), *p* = 0.98 | 1.04 (0.91-1.21), *p* = 0.55 | 1.03 (0.87-1.21), *p* = 0.75 | 1.09 (0.92-1.28), *p* = 0.33 |
| **Rectum** | 1.00 (0.81-1.22), *p* = 0.98 | 1.03 (0.84-1.26), *p* = 0.80 | 1.06 (0.87-1.27), *p* = 0.59 | 1.10 (0.91-1.32), *p* = 0.34 |
| **Non-Hodgkin’s’ Lymphoma** | 1.10 (0.92-1.32), *p* = 0.29 | 1.11 (0.92-1.33), *p* = 0.26 | 0.95 (0.79-1.15), *p* = 0.61 | 0.96(0.79-1.16), *p* = 0.67 |
| **Kidney** | 1.04 (0.80-1.34), *p* = 0.77 | 1.05 (0.81-1.35), *p* = 0.74 | 0.90 (0.73-1.12), *p* = 0.35 | 0.90 (0.73-1.11), *p* = 0.34 |
| **Pancreas** | 0.95 (0.75-1.20), *p* = 0.65 | 0.96 (0.75-1.21), *p* = 0.70 | 1.14 (0.87-1.49), *p* = 0.34 | 1.16 (0.88-1.51), *p* = 0.29 |
| **Bladder** | 0.84 (0.60-1.19), *p* = 0.50 | 0.88 (0.62-1.24), *p* = 0.47 | 0.93 (0.74-1.18), *p* = 0.55 | 0.95 (0.75-1.20), *p* = 0.66 |
| **Oesophagus** | 0.79 (0.57-1.08), *p* = 0.01 | 0.78 (0.57-1.08), *p* = 0.13 | 0.69 (0.56-0.87), *p* = 0.001 | 0.71 (0.57-0.89), *p* = 0.003 |

CI = Confidence Interval, HR = Hazard Ratio *^a^ When hormone replacement therapy (ever vs never) was added to the model, the results for pre-menopausal breast cancer were HR= 1.12 (95% CI, 1.00-1.24), p = 0.042, post-menopausal breast cancer HR = 1.08 (95% CI = 1.01-1.16), p = 0.02, and endometrial cancer HR = 1.08 (95% CI, 0.94-1.25), p = 0.30.

**Supplementary Table 4:** Hazard ratios by birth cohorts for cancer sites where birth cohort interactions were identified

| **Birth Cohort** | **Women** | **Men** | |
| --- | --- | --- | --- |
|  | **Kidney cancer**  **N (Number of cases)**  **HR (95% CI)** | **Lung cancer**  **N (Number of cases)**  **HR (95% CI)** | **Malignant melanomas of the skin**  **N (Number of cases)**  **HR (95% CI)** |
| **1939-1944** | 42,446 (124)  1.49 (0.92-2.42) | 35,834 (604)  1.18 (0.94-1.48) | 35,834 (317)  1.56 (1.01-2.22) |
| **1945-1949** | 43,079 (94)  1.17 (0.71-1.92) | 32,500 (277  1.21 (0.88-1.65) | 32,500 (222)  1.20 (0.88-1.65) |
| **1950-1954** | 34,443 (50)  0.52 (0.29-0.92) | 25,364 (139)  0.84 (0.55-1.18) | 25,364 (147)  1.13 (0.76-1.68) |
| **1955-1959** | 31,286 (26)  2.91 (0.99-8.56) | 22,871 (59)  0.50 (0.29-0.85) | 22,871 (91)  1.28 (0.79-2.09) |
| **1960-1972** | 39,863 (27)  0.52 (0.23-1.18) | 31,429 (28)  1.57 (0.68-3.64) | 31,429 (104)  0.83 (0.56-1.24) |

N = Number of participants, HR = Hazard Ratio, CI = Confidence Interval. The *p* value for the log-likelihood test between a model with and without birth cohort interaction for kidney cancer in women was *p* = 0.003. In men, lung cancer *p* = 0.02 and Malignant melanomas of the skin *p* = 0.04.

**Supplementary Figure 1:** Age group-specific hazard ratios for Non-Hodgkin's Lymphoma and Kidney cancer in women.

**
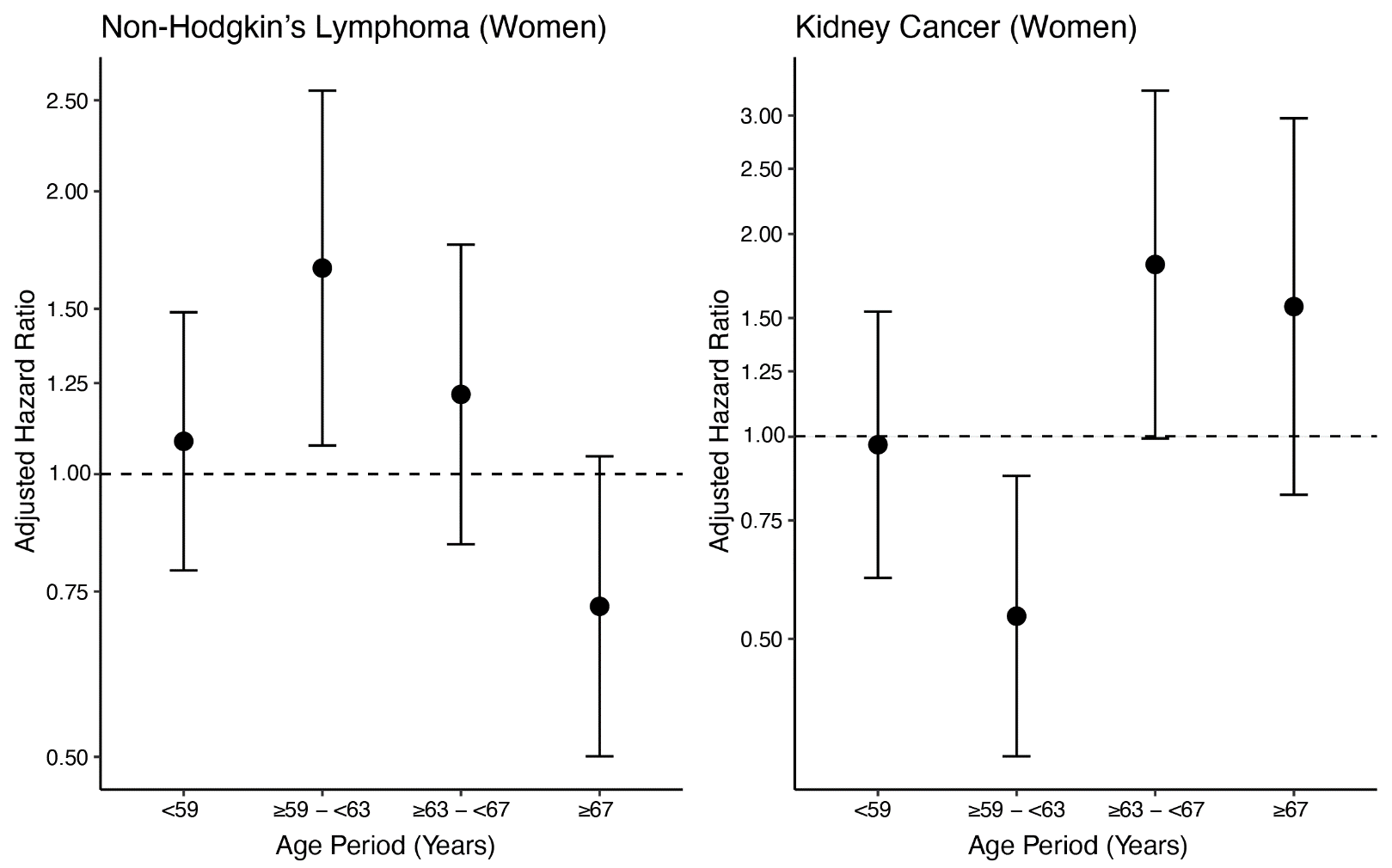
**
